# Transfusion strategies in patients with acute coronary syndrome and anemia: a meta-analysis

**DOI:** 10.1186/s43044-022-00252-2

**Published:** 2022-03-21

**Authors:** Usama Nasir, Tayyab Ali Waheed, Keerat Rai Ahuja, Charnjeet Singh Sandhu, Muhammad Ameen, Earl J. Hope

**Affiliations:** 1 Department of Internal Medicine, Reading Hospital-Tower Health, Sixth and Spruce Streets, West Reading, PA 19612 USA; 2 Department of Cardiology, Reading Hospital-Tower Health, Reading, PA USA; 3 Department of Cardiology and Interventional Cardiology, Reading Hospital-Tower Health, Reading, PA USA

**Keywords:** ACS, Transfusion, Restrictive versus liberal

## Abstract

**Background:**

Anemia is a known risk factor for ischemic heart disease and serves as an independent predictor of major adverse cardiovascular events (MACE) in patients with acute coronary syndrome (ACS). This meta-analysis pools data from randomized controlled trials (RCTs) to better define hemoglobin (Hb) thresholds for transfusion in this setting.

**Results:**

MEDLINE, EMBASE, and Cochrane databases were searched using the terms “Acute Coronary Syndrome” AND “Blood Transfusion” including their synonyms. A total of three randomized controlled trials were included. Restrictive transfusion strategy (RTS) was defined as transfusing for Hb ≤ 8 g/dl with a post-transfusion goal of 8 to 10 g/dl. Liberal transfusion strategy (LTS) was defined as Hb ≤ 10 g/dl and post-transfusion goal of at least 11 g/dl. The primary end point was 30-day mortality. Secondary outcomes included recurrent ACS events, new or worsening CHF within 30 days, and major adverse cardiac events (MACE). The primary analytic method used was random effects model. Out of 821 patients, 400 were randomized to LTS, and 421 to RTS. Mean age was 70.3 years in RTS versus 76.4 in LTS. There was no statistically significant difference for 30-day mortality in LTS compared to RTS [odds ratio (OR) 1.69; 95% CI 0.35 to 8.05]. Similarly, there was no difference in MACE (OR 0.74; 95% CI 0.21 to 2.63), CHF (OR 0.82; 95% CI 0.18 to 3.76), or the incidence of recurrent ACS (OR 1.21; 95% CI 0.49 to 2.95).

**Conclusions:**

In the setting of ACS, there is no difference between LTS and RTS for the outcomes of mortality, MACE, recurrent ACS, or CHF at 30 days. Further evidence in the form of high-quality RCTs are needed to compare RTS and LTS.

**Supplementary Information:**

The online version contains supplementary material available at 10.1186/s43044-022-00252-2.

## Background

Anemia is a known risk factor for ischemic heart disease and serves as an independent predictor of major adverse cardiovascular events in patients with acute coronary syndrome (ACS) [1, 2]. Anemic patients presenting in the setting of ACS should therefore be triaged early towards the need for a blood transfusion to maintain hemoglobin (Hb) levels above a certain threshold to prevent adverse outcomes. This threshold has not been clearly defined in the literature due to vast heterogeneity in data. The 2014 guidelines from the American Heart Association and American College of Cardiology discourage against routine blood transfusion in hemodynamically stable patients with NSTE-ACS and hemoglobin levels greater than 8 g/dl [3]

To date, only three randomized clinical trials (RCTs) have compared a liberal transfusion strategy (LTS) with a restrictive transfusion strategy (RTS) in this clinical setting [4–6]. While the first trial favored RTS, the second favored LTS. Both these trials were limited by their sample size. With the recent results from the REALITY trial, it is imperative to perform a meta-analysis for improved statistical power to better define these thresholds.

## Methods

### Search strategy and study selection

The design of the meta-analysis is based on the research question of interest, which is whether there is any difference in outcomes between RTS and LTS in the setting of ACS.

Research databases including Medline, EMBASE, and Cochrane central registry of controlled trials were queried since inception through December 1, 2021. Relevant terms and their synonyms, including but not limited to Acute Coronary Syndrome” OR “ACS” OR “Myocardial infarction,” AND “Blood Transfusion,” were used in different combinations.

Only randomized clinical trials of adult human subjects published as full manuscripts in English were included. Studies were limited to those comparing a restrictive versus liberal transfusion strategy in the setting of acute coronary syndrome only. Screening and data extraction were performed by two independent authors (UN and TAW) with discrepancies resolved by a third author (KRA). Selected studies were reviewed, and both qualitative and quantitative data were extracted. PRISMA guidelines were used for abstracting data and assessing data quality and validity [7]. Three studies were included in the final analysis.

### Defining main outcomes and measures

Restrictive transfusion strategy (RTS) was defined as transfusing for Hb ≤ 8 g/dl with a post-transfusion goal of 8 to 10 g/dl. Liberal transfusion strategy (LTS) was defined as transfusing for Hb ≤ 10 g and post-transfusion goal of at least 11 g/dl.

The primary outcome of the study was 30-day mortality in LTS compared to RTS. Secondary outcomes included the incidence of CHF, incidence of recurrent ACS, and MACE, which was defined as the composite outcome of 30-day all-cause mortality, MI, and CHF.

### Statistical analysis

Both random- and fixed-effects model of Mantel–Haenszel were used to calculate pooled odds ratio (OR) and the corresponding 95% confidence interval (CI) for primary and secondary outcomes. Heterogeneity was assessed using the Higgins *I*^2^ statistic, with values < 25% considered as low and > 75% as indicators for high heterogeneity. Sensitivity analysis was performed excluding individual trials to check consistency of the results.

Two reviewers separately evaluated the risk of bias in individual studies according to the Cochrane risk-of-bias tool for randomized trials, version 2. Risk of bias is reported at the trial level as the final aggregate of individual biases. Publication bias was assessed by funnel plots. Analysis was performed using R Studio version 1.246. This data is provided in the Additional file 1.

## Results

The study included 821 patients; 400 patients were randomized to LTS, and 421 to RTS. Mean age was 70.3 years in RTS versus 76.4 years in LTS. ACS presentations ranged from ST elevation MI, non-ST elevation MI, unstable angina, and stable coronary artery disease undergoing coronary catheterization. Major exclusion criteria included hemodynamic instability and the receipt of blood transfusion within the previous 30 days. Baseline study and patient characteristics are tabulated in detail in Tables 1 and 2.

**Table 1:** Baseline study characteristics

Study	LTS (*n*)	RTS (*n*)	Definition of RTS and LTS	Key inclusion criteria	Key exclusion criteria	Types of ACS	Follow-up duration	Outcomes of Interest
Cooper et al./CRIT 2011 [4]	21	24	LTS: hematocrit < 30% with post-transfusion goal of 30–33% RTS: hematocrit < 24% with post-transfusion goal 24% to 27%	AMI (ischemic-type chest discomfort lasting ≥ 30 min and associated with a creatine kinase-MB (CK-MB) or cardiac troponin level above the upper limit of normal. Hematocrit ≤ 30% within 72 h of symptom onset	Age < 21; non-coronary cause for clinical syndrome; active bleeding; RBC transfusion within 7 days of enrollment; imminent death; pregnancy	STE/NSTE	1 month	All-cause mortality; in-hospital mortality; recurrent MI/ACS, 30-day mortality
Carson et al. 2013 [5]	55	55	LTS: < 10 g/dl with post-transfusion goal > 10 g/dl RTS: < 8 g/dl or symptomatic for post-transfusion goal > 8 g/dl	Age ≥ 18; STEMI, NSTEMI, unstable angina, stable CAD undergoing cardiac catheterization; Hb < 10 g/dl at the time of random allocation	Hgb > 10; symptoms of anemia at the time of randomization; Cardiac surgery within 30 days; severe illness; Ventilated/intubated; hemodynamic instability	STE/NSTE/stable angina	1 month	All-cause mortality; in-hospital mortality; recurrent MI/ACS; 30-day mortality
Ducrocq et al. 2021/REALITY [6]	324	342	LTS: ≤ 10 g/dl, with post-transfusion goal ≥ 11 g/dl RTS: ≤ 8 g/dl, with post-transfusion goal 8–10 g/dl	Age ≥ 18; AMI (with or without ST-segment elevation with a combination of ischemic symptoms occurring in the 48 h before admission and elevation of biomarkers, and Hb 7–10 g/dl	Shock; MI occurring after PCI or CABG; life-threatening or massive ongoing bleeding; blood transfusion in the past 30 days; malignant hematologic disease	STE/NSTE	1 month	All-cause mortality; in-hospital mortality; recurrent MI/ACS; 30-day mortality

*LTS* liberal transfusion strategy, *RTS* restrictive transfusion strategy, *AMI* acute myocardial infarction, *MI* myocardial infarction, *ACS* acute coronary syndrome, *STE* ST elevation, *NSTE* non-ST elevation, *CAD* coronary artery disease, *CK* creatinine kinase

**Table 2:** Baseline patient characteristics

Study	Male (*n*/%)	Mean age (years)	HTN (*n*/%)	Prior MI (*n*/%)	DM (*n*/%)	Prior CABG (*n*/%)	Prior PCI (*n*/%)	Presenting with STEMI (*n*/%)	Presenting with NSTEMI (*n*/%)
Cooper et al./CRIT 2011 [4]	R: 13/54 L:10/48	R: 70.3 L: 76.4	R: 18/75 L: 19/91	R: 15/63 L: 16/76	R: 13/54 L: 17/81	R: 4/17 L: 6/29	R: 6/25 L: 5/24	R: 11/46 L: 7/33	R: 13/54 L: 14/67
Carson et al. 2013 [5]	R: 27/49.1 L: 28/50.9	R: 74.3 L: 67.3	R: 45/81.8 L: 47/85.5	R: 36/65.5 L: 38/69.1	R: 29/52.7 L: 34/61.8	R: 18/32.7 L: 16/29.1	R: 22/40 L: 24/43.6	R: 16/29.1 L: 17/30.1	R: 26/47.3 L: 21/38.2
Ducrocq et al. 2021/REALITY [6]	R: 201/58.8 L: 184/56.8	R: 78 L: 76	R: 272/79.5 L: 256/79.0	R: 189/55.3 L: 201/62.0	R: 176/51.5 L: 158/48.8	R: 44/12.9 L: 42/13.0	R: 114/33.3 L: 111/34.3	R: 108/31.6 L: 93/28.7	R: 234/68.4 L: 231/71.3

*HTN* hypertension, *DM* diabetes mellitus, *MI* myocardial infarction, *PCI* percutaneous coronary intervention, *R* restrictive transfusion, *L* liberal transfusion

**Fig. 1:**
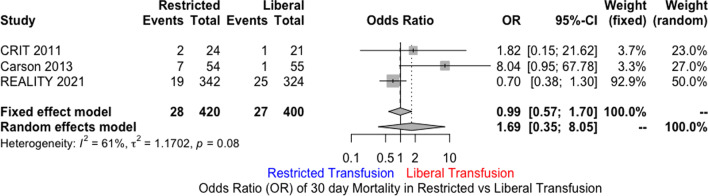
Odds ratio of 30 day all-cause mortality. Values present are OR with 95% confidence interval, *n*, or percentage %. Restricted ≤ 8 g/dl; Liberal ≤ 10 g/dl

**Fig. 2:**
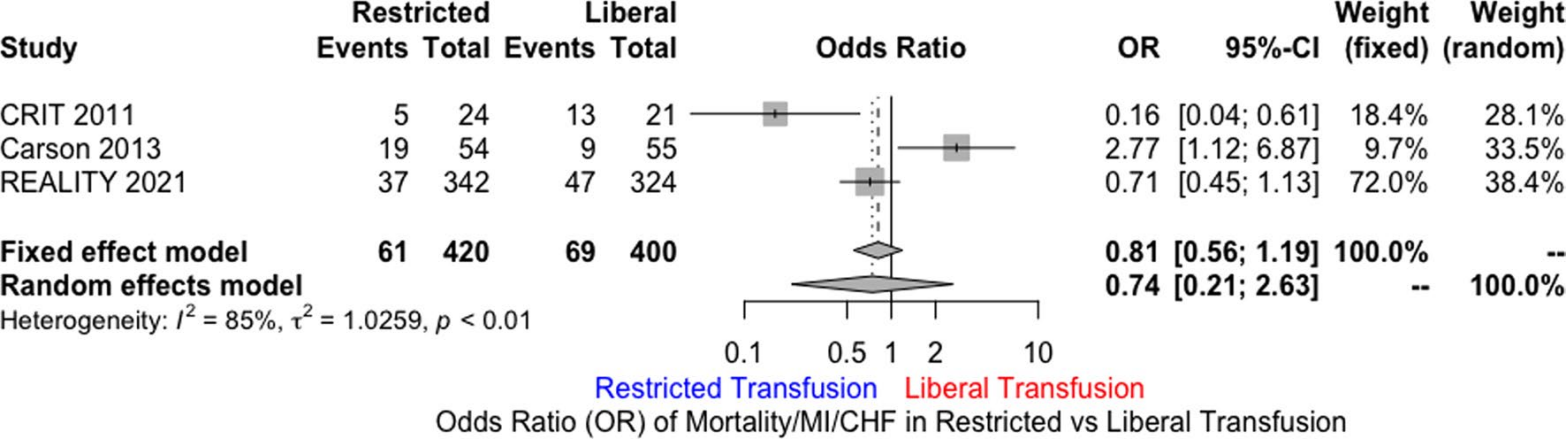
MACE at 30 days defined as a composite outcome of 30 day all-cause mortality, MI, and CHF. Values present are OR with 95% confidence interval, *n*, or percentage %. Restricted ≤ 8 g/dl; Liberal ≤ 10 g/dl

**Fig. 3:**
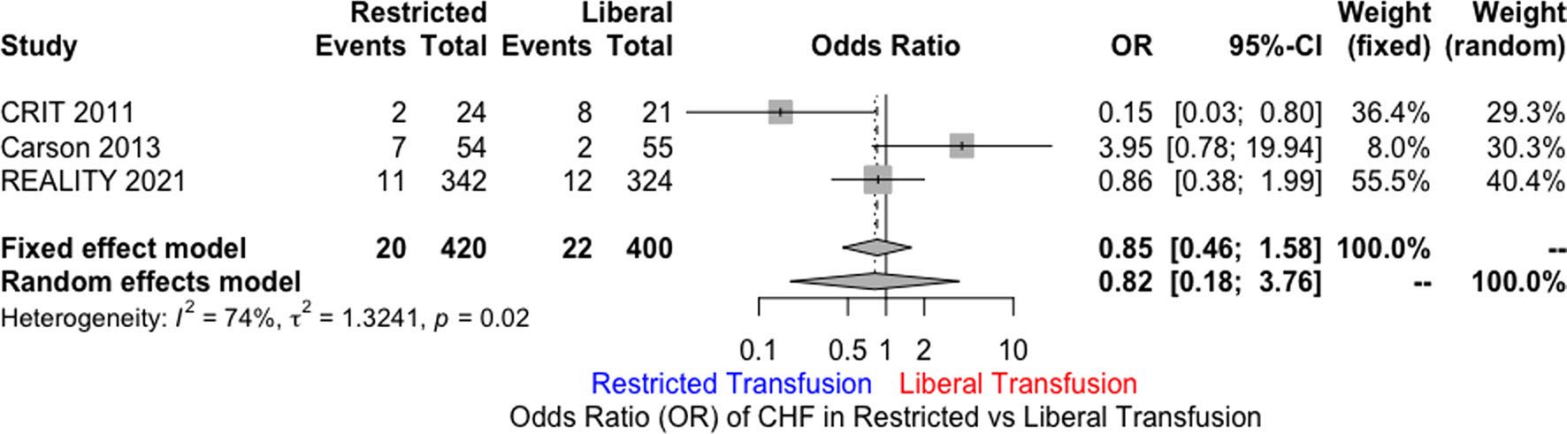
Odds ratio of new or worsening congestive heart failure at 30 days. Values present are OR with 95% confidence interval, *n*, or percentage %. Restricted ≤ 8 g/dl; Liberal ≤ 10 g/dl

**Fig. 4:**
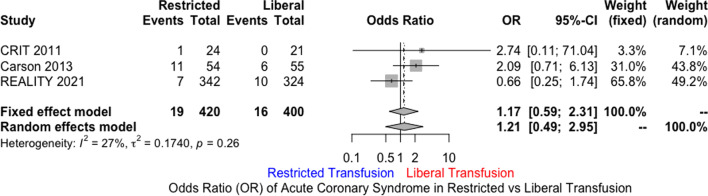
Odds ratio of recurrent acute coronary syndrome at 30 days. Values present are OR with 95% confidence interval, *n*, or percentage %. Restricted ≤ 8 g/dl; Liberal ≤ 10 g/dl

**Fig. 5:**
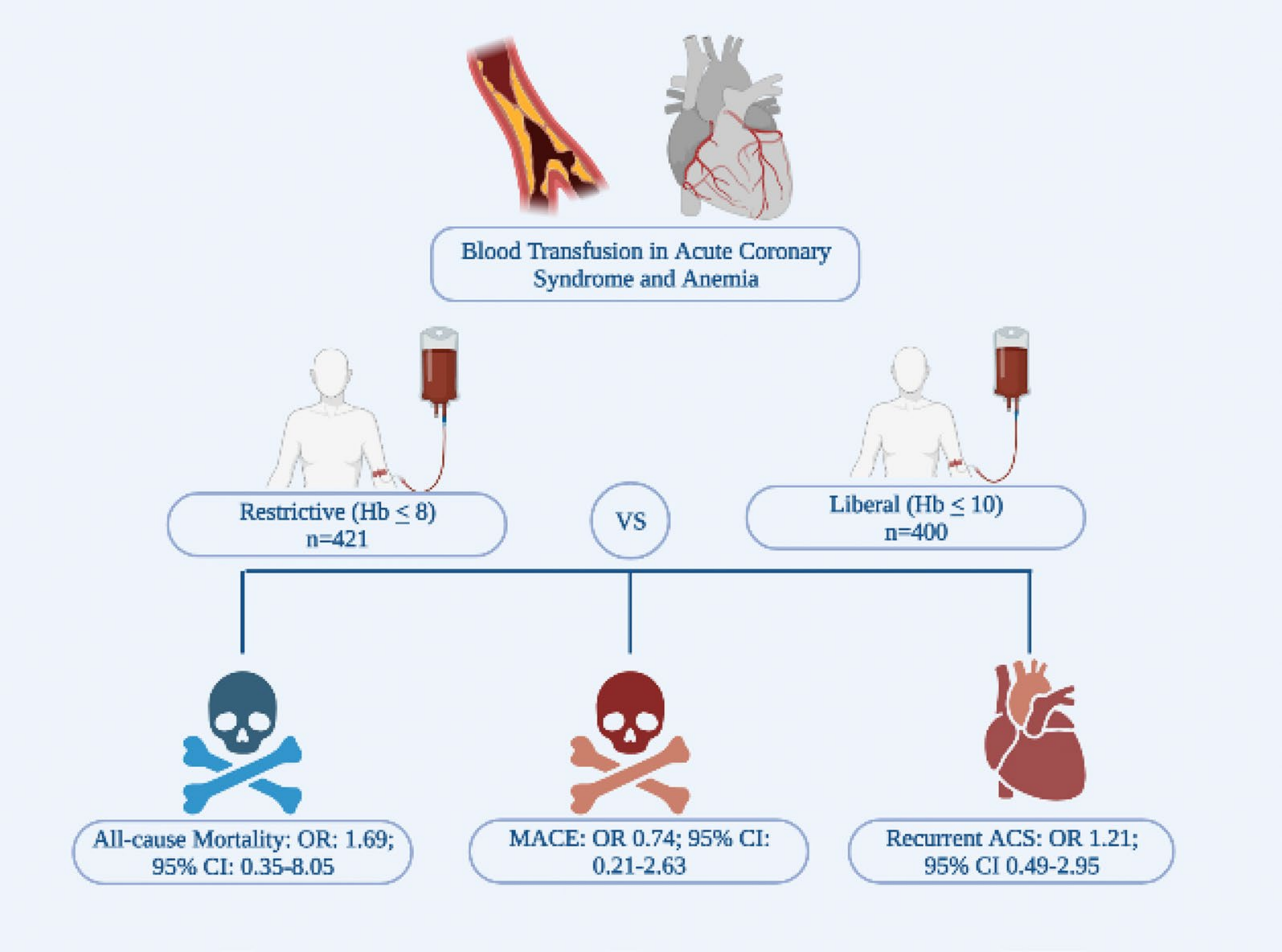
Graphical abstract. *OR* odds ratio, *Hb* Hemoglobin

There was no statistically significant difference for 30-day mortality in LTS compared to RTS [odds ratio (OR) 1.69; 95% CI 0.35 to 8.05; *I*^2^ 61%) (Fig. 1). Similarly, there was no significant difference in MACE (OR 0.74; 95% CI 0.21 to 2.63; *I*^2^ 85%) (Fig. 2), CHF (OR 0.82; 95% CI 0.18 to 3.76; *I*^2^ 74%), and the incidence of recurrent acute coronary syndrome (OR 1.21; 95% CI 0.49 to 2.95; *I*^2^ 27%) (Figs. 3, 4, 5). Given the high heterogeneity, sensitivity analysis was performed excluding individual trials which showed consistent results.

## Discussion

This meta-analysis is the first to pool randomized controlled trials in order to better study the association between transfusion thresholds and outcomes in ACS. The results highlight several important findings. Transfusing for Hb less than or equal to 10 g/dl appears to offer no benefit in 30 day mortality compared to transfusing below a threshold equal to or less than 8 g/dl. Similarly, there appears to be no added benefit of a liberal transfusion strategy in any of the studied outcomes which include MACE, recurrence of ACS, or incidence of CHF.

In a previous meta-analysis by Garfinkle et al. limited to observational studies, transfusion below 8 g/dl had beneficial or neutral effects compared to harmful effects above 11 g/dl [8]. Wang et al. report in their meta-analysis of both observational studies and RCTs, a higher risk of 30 day mortality (RR = 1.21, 95% CI 1.01–1.45) in the restrictive group compared to the liberal group. However, their study included a majority of patients with underlying coronary artery disease, and patients undergoing non-cardiac surgery [9].

Multiple studies have compared the outcomes of blood transfusion versus no blood transfusion in the ACS setting and reported higher mortality with blood transfusions [10–12]. Similarly multiple studies have also compared a restrictive and liberal transfusion strategy in patients with cardiovascular disease in the setting of surgery and critical illness and favored lower thresholds [13]. However, there remains paucity of high quality studies comparing restrictive and liberal transfusion strategies with set thresholds in the setting of ACS.

The current meta-analysis includes patients with STEMI, NSTEMI, and unstable angina. In our included studies, the first of the trials favored RTS, with higher rates of CHF reported in LTS [4]. Carson et al. later provided support for LTS owing to lower CV mortality [5]. These trials, however, were limited by their small sample sizes. The recently conducted REALITY trial provided a larger sample size and showed that RTS was non-inferior to LTS [6]. The overall results of our analysis are in contrast to the meta-analysis by Wang et al. [9] and in agreement with the REALITY trial, i.e., in the ACS settings, there is no statistically significant difference in outcomes between RTS and LTS.

The 2014 American Heart Association/American College of Cardiology guidelines do not recommend routine blood transfusion in hemodynamically stable patients with NSTE-ACS and hemoglobin levels greater than 8 g/dl (Class III, level of evidence C) [14]. Similarly, the 2020 European Society of Cardiology recommendations for the management of NSTE-ACS include leaning away from RBC transfusions for Hb above 8 g/dl or hematocrit greater than 25% (Class IIb, level of evidence C) [15].

Our study has multiple limitations. Firstly, the study was limited by the sample size with the predominant contribution from the REALITY trial, while the other two included trials significantly smaller sample sizes. Secondly, in the study by Carson et al. only patients with symptomatic anemia received transfusion in the restrictive arm compared to other studies included in this meta-analysis. Thirdly, due to lack of availability, study level data were used instead of patient level data; therefore, meta-regression for specific variables could not be performed.

## Conclusions

This study shows no difference in major outcomes including 30-day mortality while comparing a liberal versus restrictive transfusion strategy in the setting of ACS. Further, high-quality randomized controlled trials are required to better compare transfusion thresholds in the setting of ACS. The ongoing MINT trial (NCT02981407) will provide further evidence in this regard.

## Supplementary Information

**Additional file 1. Table 1:** Electronic Database Search Strategy. **Figure 1:** PRISMA Diagram. **Table 2**: PRISMA checklist. **Table 3**: Risk of bias assessment of trials. **Figure 2:** Funnel plot: Mortality. **Figure 3:** Funnel plot MACE. **Figure 4:** Funnel plot CHF. **Figure 5:** Funnel plot recurrent MI..

## Data Availability

Not applicable.

## Abbreviations

RTS: Restrictive transfusion strategy
LTS: Liberal transfusion strategy
Hb: Hemoglobin
ACS: Acute coronary syndrome
MACE: Mean adverse cardiovascular events
