# Correction: Transfusion strategies in patients with acute coronary syndrome and anemia: a meta-analysis

**DOI:** 10.1186/s43044-022-00262-0

**Published:** 2022-04-04

**Authors:** Usama Nasir, Tayyab Ali Waheed, Keerat Rai Ahuja, Charnjeet Singh Sandhu, Muhammad Ameen, Earl J. Hope

**Affiliations:** 1 Department of Internal Medicine, Reading Hospital-Tower Health, Sixth and Spruce Streets, West Reading, PA 19612 USA; 2 Department of Cardiology, Reading Hospital-Tower Health, Reading, PA USA; 3 Department of Cardiology and Interventional Cardiology, Reading Hospital-Tower Health, Reading, PA USA

## Correction to: The Egyptian Heart Journal (2022) 74:17 https://doi.org/10.1186/s43044-022-00252-2

Following publication of the original article [1], the authors identified a typesetting error in Table 1. The correctly formatted table is given below. The original article [1] has been corrected.

**Table 1:** Baseline study characteristics

Study	LTS (*n*)	RTS (*n*)	Definition of RTS and LTS	Key inclusion criteria	Key exclusion criteria	Types of ACS	Follow-up duration	Outcomes of interest
Cooper et al./CRIT 2011 [4]	21	24	LTS: hematocrit < 30% with post-transfusion goal of 30–33% RTS: hematocrit < 24% with post-transfusion goal 24–27%	AMI (ischemic-type chest discomfort lasting ≥ 30 min and associated with a creatine kinase-MB (CK-MB) or cardiac troponin level above the upper limit of normal. Hematocrit ≤ 30% within 72 h of symptom onset	Age < 21; non-coronary cause for clinical syndrome; active bleeding; RBC transfusion within 7 days of enrollment; imminent death; pregnancy	STE/NSTE	1 month	All-cause mortality; in-hospital mortality; recurrent MI/ACS, 30-day mortality
Carson et al. 2013 [5]	55	55	LTS: < 10 g/dl with post-transfusion goal > 10 g/dl RTS: < 8 g/dl or symptomatic for post-transfusion goal > 8 g/dl	Age ≥ 18; STEMI, NSTEMI, unstable angina, stable CAD undergoing cardiac catheterization; Hb < 10 g/dl at the time of random allocation	Hgb > 10; symptoms of anemia at the time of randomization; cardiac surgery within 30 days; severe illness; Ventilated/intubated; hemodynamic instability	STE/NSTE/stable angina	1 month	All-cause mortality; in-hospital mortality; recurrent MI/ACS; 30-day mortality
Ducrocq et al. 2021/REALITY [6]	324	342	LTS: ≤ 10 g/dl, with post-transfusion goal ≥ 11 g/dl RTS: ≤ 8 g/dl, with post-transfusion goal 8–10 g/dl	Age ≥ 18; AMI (with or without ST-segment elevation with a combination of ischemic symptoms occurring in the 48 h before admission and elevation of biomarkers, and Hb 7–10 g/dl	Shock; MI occurring after PCI or CABG; life-threatening or massive ongoing bleeding; blood transfusion in the past 30 days; malignant hematologic disease	STE/NSTE	1 month	All-cause mortality; in-hospital mortality; recurrent MI/ACS; 30-day mortality

*LTS* liberal transfusion strategy, *RTS* restrictive transfusion strategy, *AMI* acute myocardial infarction, *MI* myocardial infarction, *ACS* acute coronary syndrome, *STE* ST elevation, *NSTE* non-ST elevation, *CAD* coronary artery disease, *CK* creatinine kinase
