# Supplementary material for: Transfusion strategies in patients with acute coronary syndrome and anemia: a meta-analysis
Source: Egypt Heart J. 2022 Mar 21;74:17. doi: 10.1186/s43044-022-00252-2 (PMC8938537; doi:10.1186/s43044-022-00252-2)
Supplement: Supplementary file 1 — Additional file 1. Table 1: Electronic Database Search Strategy. Figure 1: PRISMA Diagram. Table 2: PRISMA checklist. Table 3: Risk of bias assessment of trials. Figure 2: Funnel plot: Mortality. Figure 3: Funnel plot MACE. Figure 4: Funnel plot CHF. Figure 5: Funnel plot recurrent MI. [file 43044_2022_252_MOESM1_ESM.docx]

Supplemental content

**Transfusion Strategies in Patients with Acute Coronary Syndrome and Anemia: A Meta-Analysis**

Usama Nasir MD, Tayyab Ali Waheed MD, Keerat Rai Ahuja MD, FACP, Charnjeet Singh Sandhu MD, Muhammad Ameen MD, Earl J Hope MD, FACC, FSCAI

1. **Search strategy**
2. **PRISMA diagram**
3. **PRISMA checklist**
4. **Risk of bias assessment**
5. **Funnel plot**

| Database | Search strategy | Search Results |
| --- | --- | --- |
| PubMed/MEDLINE | (Myocardial Infarction[MESH] OR “myocardial infarction” OR “cardiovascular stroke” OR “myocardial infarct” OR “heart attack” OR Unstable Angina[MESH] OR “unstable angina” OR “unstable angina pectoris” OR “angina at rest” OR “preinfarction angina” OR “myocardial preinfarction syndrome” OR Acute Coronary Syndrome[MESH] OR “acute coronary syndrome” OR ST Elevation Myocardial Infarction[MESH] OR “ST Elevated Myocardial Infarction” OR “ST segment myocardial infarction” OR STEMI OR Non-ST Elevated Myocardial Infarction[MESH] OR “Non-ST Elevated Myocardial Infarction” OR “Non ST Elevated Myocardial Infarction” OR NSTEMI OR “Non ST Elevation Myocardial Infarction” OR “Non-ST Elevation Myocardial Infarction”) AND (Blood Transfusion[MESH] OR “blood transfusion” OR “liberal blood transfusion” OR “restrictive blood transfusion” OR “restrictive transfusion” OR “liberal transfusion” OR “conservative transfusion”) AND (randomized controlled trial[Publication Type] OR (randomized[Title/Abstract] AND controlled[Title/Abstract] AND trial[Title/Abstract])) | 160 |
| EMBASE | ('st segment elevation myocardial infarction'/exp OR 'st elevated mi' OR 'st elevated myocardial infarction' OR 'st elevation mi' OR 'st elevation myocardial infarction' OR 'st segment elevated myocardial infarction' OR 'st segment elevation mi' OR 'st segment elevation heart infarction' OR 'st segment elevation myocardial infarction' OR 'stemi' OR 'heart infarction'/exp OR 'cardiac infarct' OR 'cardiac infarction' OR 'cardial infarct' OR 'heart attack' OR 'heart infarct' OR 'heart infarction' OR 'heart micro infarction' OR 'heart muscle infarction' OR 'infarction, heart' OR 'myocardial infarct' OR 'myocardial infarction' OR 'myocardium infarct' OR 'myocardium infarction' OR 'premonitory infarction sign' OR 'second heart attack' OR 'subendocardial infarction' OR 'transmural cardiac infarction' OR 'transmural heart infarction' OR 'transmural infarction, heart' OR 'non st segment elevation myocardial infarction'/exp OR 'nstemi' OR 'non st elevated mi' OR 'non st elevated myocardial infarction' OR 'non st elevation mi' OR 'non st elevation myocardial infarction' OR 'non st segment elevated myocardial infarction' OR 'non st segment elevation mi' OR 'non st segment elevation heart infarction' OR 'non st segment elevation myocardial infarction' OR 'non stemi' OR 'non-st elevated myocardial infarction' OR 'acute coronary syndrome'/exp OR 'acute coronary syndrome' OR 'acute coronary syndromes') AND ('blood transfusion'/exp OR 'blood exchange' OR 'blood infusion' OR 'blood replacement' OR 'blood retransfusion' OR 'blood transfusion' OR 'hemotherapy' OR 'multitransfusion' OR 'polytransfusion' OR 'retransfusion' OR 'transfusion blood' OR 'transfusion therapy' OR 'transfusion, blood' OR 'liberal transfusion'/exp OR 'restrictive transfusion'/exp OR 'conservative transfusion') AND [randomized controlled trial]/lim | 496 |
| Cochrane CENTRAL | ( (ZU "myocardial infarction" OR "myocardial infarction" OR "heart attack" OR ZU "st elevation myocardial infarction" OR STEMI OR ZU "non-st elevated myocardial infarction" OR NSTEMI OR ZU "acute coronary syndrome" OR "acute coronary syndrome") ) AND ( (ZU "blood transfusion" OR "blood transfusion" OR "liberal transfusion" OR "restrictive transfusion" OR "conservative transfusion") ) | 358 |

**Table 1: Electronic Database Search Strategy**

MEDLINE = Medical Literature Analysis and Retrieval System Online; CENTRAL = Central Register of Controlled Trials; EMBASE = Excerpta Medica Database.

**Figure 1: PRISMA Diagram**

**Identification of studies via databases and registers**

Records identified from:

EMBASE (n = 496)

PubMed (n = 160)

Cochrane Central (n = 358)

Records removed *before screening*:

Duplicate records removed (n = 3)

**Identification**

Records screened

(n = 1001 )

Records excluded

(n = 976 )

Reports sought for retrieval

(n =25 )

**Screening**

Reports assessed for eligibility

(n = 25 )

Reports excluded:

Reason 1 : Non Randomized controlled trial

Reason 2 : Transfusion thresholds not clearly defined

Reason 3 : Non comparative studies

Studies included in review

(n = 3 )

Reports of included studies

(n = 3 )

**Included**

| **Section/topic** | **#** | **Checklist item** | **Reported on page #** |
| --- | --- | --- | --- |
| **TITLE** | | |  |
| Title | 1 | Identify the report as a systematic review, meta-analysis, or both. | Title page |
| **ABSTRACT** | | |  |
| Structured summary | 2 | Provide a structured summary including, as applicable: background; objectives; data sources; study eligibility criteria, participants, and interventions; study appraisal and synthesis methods; results; limitations; conclusions and implications of key findings; systematic review registration number. | 1,2 |
| **INTRODUCTION** | | |  |
| Rationale | 3 | Describe the rationale for the review in the context of what is already known. | 3 |
| Objectives | 4 | Provide an explicit statement of questions being addressed with reference to participants, interventions, comparisons, outcomes, and study design (PICOS). | 3 |
| **METHODS** | | |  |
| Protocol and registration | 5 | Indicate if a review protocol exists, if and where it can be accessed (e.g., Web address), and, if available, provide registration information including registration number. | - |
| Eligibility criteria | 6 | Specify study characteristics (e.g., PICOS, length of follow-up) and report characteristics (e.g., years considered, language, publication status) used as criteria for eligibility, giving rationale. | 4,5 |
| Information sources | 7 | Describe all information sources (e.g., databases with dates of coverage, contact with study authors to identify additional studies) in the search and date last searched. | 4,5 |
| Search | 8 | Present full electronic search strategy for at least one database, including any limits used, such that it could be repeated. | 4,5 |
| Study selection | 9 | State the process for selecting studies (i.e., screening, eligibility, included in systematic review, and, if applicable, included in the meta-analysis). | 4,5 |
| Data collection process | 10 | Describe method of data extraction from reports (e.g., piloted forms, independently, in duplicate) and any processes for obtaining and confirming data from investigators. | 4,5 |
| Data items | 11 | List and define all variables for which data were sought (e.g., PICOS, funding sources) and any assumptions and simplifications made. | 4,5 |
| Risk of bias in individual studies | 12 | Describe methods used for assessing risk of bias of individual studies (including specification of whether this was done at the study or outcome level), and how this information is to be used in any data synthesis. | 4,5 |
| Summary measures | 13 | State the principal summary measures (e.g., risk ratio, difference in means). | 4,5 |
| Synthesis of results | 14 | Describe the methods of handling data and combining results of studies, if done, including measures of consistency (e.g., I^2^) for each meta-analysis. | 4,5 |

| **Section/topic** | **#** | **Checklist item** | **Reported on page #** |
| --- | --- | --- | --- |
| Risk of bias across studies | 15 | Specify any assessment of risk of bias that may affect the cumulative evidence (e.g., publication bias, selective reporting within studies). | 4,5 |
| Additional analyses | 16 | Describe methods of additional analyses (e.g., sensitivity or subgroup analyses, meta-regression), if done, indicating which were pre-specified. | na |
| **RESULTS** | | |  |
| Study selection | 17 | Give numbers of studies screened, assessed for eligibility, and included in the review, with reasons for exclusions at each stage, ideally with a flow diagram. | 4,5 |
| Study characteristics | 18 | For each study, present characteristics for which data were extracted (e.g., study size, PICOS, follow-up period) and provide the citations. | 6-10 |
| Risk of bias within studies | 19 | Present data on risk of bias of each study and, if available, any outcome level assessment (see item 12). | supplementary |
| Results of individual studies | 20 | For all outcomes considered (benefits or harms), present, for each study: (a) simple summary data for each intervention group (b) effect estimates and confidence intervals, ideally with a forest plot. | 24-28 |
| Synthesis of results | 21 | Present results of each meta-analysis done, including confidence intervals and measures of consistency. | 24-28 |
| Risk of bias across studies | 22 | Present results of any assessment of risk of bias across studies (see Item 15). | na |
| Additional analysis | 23 | Give results of additional analyses, if done (e.g., sensitivity or subgroup analyses, meta-regression [see Item 16]). | na |
| **DISCUSSION** | | |  |
| Summary of evidence | 24 | Summarize the main findings including the strength of evidence for each main outcome; consider their relevance to key groups (e.g., healthcare providers, users, and policy makers). | 14,15 |
| Limitations | 25 | Discuss limitations at study and outcome level (e.g., risk of bias), and at review-level (e.g., incomplete retrieval of identified research, reporting bias). | 15,16 |
| Conclusions | 26 | Provide a general interpretation of the results in the context of other evidence, and implications for future research. | 16 |
| **FUNDING** | | |  |
| Funding | 27 | Describe sources of funding for the systematic review and other support (e.g., supply of data); role of funders for the systematic review. | Title page |

**Table 2: PRISMA checklist**

| Table 3: Risk of bias assessment of trials | | | | | | |
| --- | --- | --- | --- | --- | --- | --- |
| Study | **Randomization** | **Deviation from defined interventions** | **Missing outcome data** | **Measurement of outcomes** | **Selective reporting** | **Overall** |
| CRIT 2011 | **Low risk** | **Low risk** | **Low risk** | **Low risk** | **Low risk** | **Low risk** |
| Carson et al 2013 | **Low risk** | **Low risk** | **Low risk** | **Low risk** | **Low risk** | **Low risk** |
| REALITY 2021 | **Low risk** | **Low risk** | **Low risk** | **Low risk** | **Low risk** | **Low risk** |

**Figure 2**: Funnel plot: Mortality

**
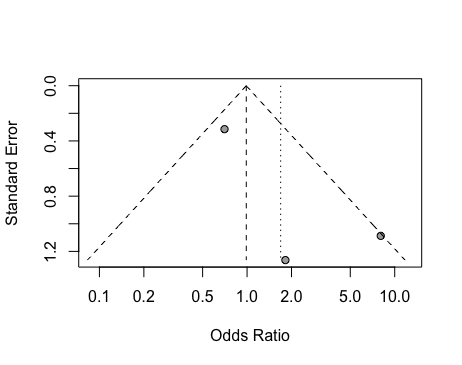
**

**Figure 3:** Funnel plot MACE

**
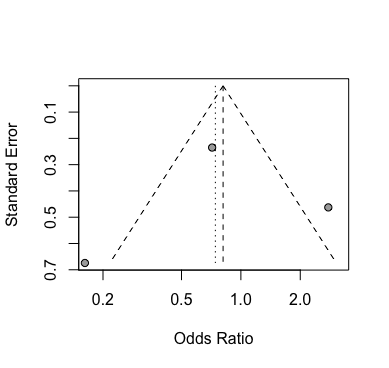
**

**Figure 4: Funnel plot CHF**

**
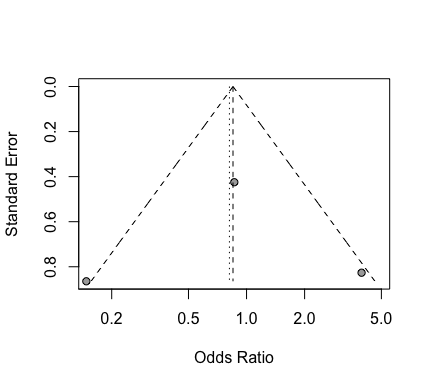
**

**Figure 5: Funnel plot recurrent MI**

**
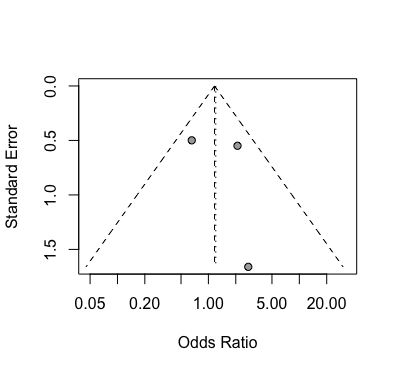
**
